# A systematic review and meta-analysis of online versus alternative methods for training licensed health care professionals to deliver clinical interventions

**DOI:** 10.1186/s12909-017-1047-4

**Published:** 2017-11-23

**Authors:** Helen Richmond, Bethan Copsey, Amanda M. Hall, David Davies, Sarah E. Lamb

**Affiliations:** 1 Warwick Clinical Trials Unit, Division of Health Sciences, Warwick Medical School, University of Warwick, Coventry, UK; 2 Centre for Rehabilitation Research, Nuffield Department of Orthopaedics, Rheumatology, and Musculoskeletal Sciences, University of Oxford, Oxford, UK; 3 The George Institute for Global Health, University of Oxford, Oxford, UK; 4 Warwick Medical School, University of Warwick, Coventry, UK

**Keywords:** Online training/learning, Internet based training/learning, E-learning, Health professionals, Continuing education, Professional development, Training, Meta-analysis, Systematic review

## Abstract

**Background:**

Online training is growing in popularity and yet its effectiveness for training licensed health professionals (HCPs) in clinical interventions is not clear. We aimed to systematically review the literature on the effectiveness of online versus alternative training methods in clinical interventions for licensed Health Care Professionals (HCPs) on outcomes of knowledge acquisition, practical skills, clinical behaviour, self-efficacy and satisfaction.

**Methods:**

Seven databases were searched for randomised controlled trials (RCTs) from January 2000 to June 2015. Two independent reviewers rated trial quality and extracted trial data. Comparative effects were summarised as standardised mean differences (SMD) and 95% confidence intervals. Pooled effect sizes were calculated using a random-effects model for three contrasts of online versus (i) interactive workshops (ii) taught lectures and (iii) written/electronic manuals.

**Results:**

We included 14 studies with a total of 1089 participants. Most trials studied medical professionals, used a workshop or lecture comparison, were of high risk of bias and had small sample sizes (range 21-183). Using the GRADE approach, we found low quality evidence that there was no difference between online training and an interactive workshop for clinical behaviour SMD 0.12 (95% CI -0.13 to 0.37). We found very low quality evidence of no difference between online methods and both a workshop and lecture for knowledge (workshop: SMD 0.04 (95% CI -0.28 to 0.36); lecture: SMD 0.22 (95% CI: -0.08, 0.51)). Lastly, compared to a manual (*n* = 3/14), we found very low quality evidence that online methods were superior for knowledge SMD 0.99 (95% CI 0.02 to 1.96). There were too few studies to draw any conclusions on the effects of online training for practical skills, self-efficacy, and satisfaction across all contrasts.

**Conclusions:**

It is likely that online methods may be as effective as alternative methods for training HCPs in clinical interventions for the outcomes of knowledge and clinical behaviour. However, the low quality of the evidence precludes drawing firm conclusions on the relative effectiveness of these training methods. Moreover, the confidence intervals around our effect sizes were large and could encompass important differences in effectiveness. More robust, adequately powered RCTs are needed.

**Electronic supplementary material:**

The online version of this article (10.1186/s12909-017-1047-4) contains supplementary material, which is available to authorized users.

## Background

The current ‘gold standard’ training for Health Care Professionals (HCPs) in clinical interventions is face-to-face workshops, supplemented with manuals and clinical supervision [1]. However, this training method places high demand on resources [2], and has limited reach due to geographical factors and restricted class sizes [3, 4]. Internet based (online) training packages are growing in popularity, offering potential advantages over alternative training methods such as widespread access in a range of settings (home, work, public spaces), personalised instruction, and regularly updated content [5–7]. Despite these advantages, there are several cited concerns including no physical presence of a teacher, learner isolation, and lack of peer support and competition [7]. These concerns are exacerbated when using online methods for developing interpersonal and high-level clinical skills, where contextual clinical reasoning underpins competence [8].

The most recent review in this area was conducted in 2008 and included 76 studies of randomised and non-randomised trials evaluating online methods versus alternative training for practicing and student HCPs. The results of their analyses suggested that there were no differences between training methods on knowledge, skills, satisfaction, and behavioural outcomes; with some interventions favouring online and others favouring the alternative [2]. The authors suggested that the lack of consistency in effects may be partly explained by the heterogeneity of learner groups, outcome measurement tools, and interventions. Therefore, the aim of this current review was to update the evidence in this rapidly developing field, and specifically focus on practicing HCPs in order to provide more contextualised information of effectiveness for this population. Specifically, we will provide a more in depth exploration of the intervention aims, content, and delivery to help guide future research in this area and provide practical implications for educators in this field.

### Aim and objectives

The aim was to systematically review the literature on the effectiveness of online methods for training licensed HCPs in a clinical intervention/topic. Our main objective was to determine the effectiveness of online versus alternative methods of training in clinical interventions/topics on knowledge and practical skills in licensed HCPs. Secondary outcomes of interest included participant satisfaction, self-efficacy, clinical behaviour, and patient outcomes.

## Methods

This systematic review and meta-analysis followed recommendations from the Cochrane Handbook for Systematic Reviews of Interventions and the PRISMA statement for systematic reviews and meta-analyses [9, 10].

### Data sources and searches

Studies were identified through an electronic search of studies from the year 2000 to 2 June 2015 in the following databases: MEDLINE (Ovid); CINAHL (Ovid); EMBASE (Ovid); AMED (Ovid); Pedro (physiotherapy evidence database); The Cochrane Library, and ASSIA. References of included studies and relevant systematic reviews were also screened. An example search strategy is provided in Additional file 1. Since internet technologies have changed dramatically from initial conception, we restricted our search dates to studies after the year 2000.

### Study selection and data extraction

#### Inclusion criteria

Studies in any language were included if they (i) were a randomised controlled trial (RCT), (ii) included licensed health care professionals (defined as a health professional that had completed their training and was certified with the relevant governing body such as the Health Professions Council in the UK), (iii) evaluated online learning to provide training in a clinical intervention (defined as an intervention carried out to improve, maintain or assess the health of a person, in a clinical situation), (iv) included a comparison arm of a training manual, a training lecture, or an interactive training workshop, and (v) assessed one of the following outcomes: HCPs satisfaction, knowledge, practical skills, self-efficacy, clinical behaviour, and patient outcomes. This series of outcomes have been used in previous reviews of training programmes [11] and are recommended as key outcomes for assessing effectiveness of educational interventions that aim to change behaviour [12]. RCTs that included an undergraduate student population were excluded, as were those studying blended learning interventions (a combination of online and face-to-face methods). We used the following definitions for our comparison interventions:

Workshop: Teaching that involved some element of collaboration and practice with peers and/or a tutor within the course/session itself, such as role play with feedback.

Lecture: the presentation of information to learners verbally with or without the use of aids such as presentation slides, with the opportunity to ask questions as the only form of interaction.

Manual: a paper or electronic training manual with no further information or interaction.

### Screening, data extraction, and quality assessment

Titles and abstracts were double screened for inclusion by two authors (HR and BC) and subsequent full texts were further double screened. Double data extraction was entered onto a standardised form and included information on: study characteristics including population (age, gender, nationality, profession and speciality), number and type of comparison interventions, outcome information (follow-up, adherence to training, measurement tool, and assessment time point), and treatment effects (numbers analysed, mean and standard deviation of treatment effects). The Template for Intervention Description and Replication (TIDieR) was used to extract data on intervention details [13]. Where outcome data was missing, we requested this information with a maximum of three emails.

Risk of bias was assessed independently by two authors (HR and BC) using the Cochrane Collaboration’s tool for assessing risk of bias [9] which included the domains (i) random sequence generation, (ii) allocation concealment, (iii) blinding of participants and personnel, (iv) blinding of outcome assessment, (v) incomplete outcome data, (vi) selective reporting, and (vii) any other source of bias. Scores from five of the items (items i, ii, iii, iv, and v) were used to rate the study as low or high risk of bias; studies rated as low on 3 or more of these items were judged to be low risk of bias.

We assessed the overall quality of the evidence using the GRADE (Grading of Recommendations, Assessment, Development and Evaluations) approach, which specifies four levels of evidence: high, moderate, low, and very low quality evidence [9]. Randomised controlled trials are considered high quality evidence. However, they can be downgraded by a maximum of three levels depending on the presence of five factors: (i) methodological quality, (ii) indirectness of evidence, (iii) inconsistency in the results, (iv) imprecision of evidence, and (v) high probability of publication bias.

### Data cleaning and missing data

Where no additional information was provided from study authors, the necessary outcome data was calculated from alternative study data where possible, for example, computing the standard deviation from the 95% confidence interval [9].

### Data synthesis

#### Meta-analyses

Between group differences were calculated from post-treatment scores and reported as standardised mean difference (SMD) with 95% confidence intervals (CIs). Where applicable, scales were reversed by subtracting the mean from the maximum score for the scale to ensure a consistent direction of effect across studies. A positive SMD represented an effect in favour of online training. Effect sizes were interpreted as: 0.2 indicating a small effect, 0.5 indicating a moderate effect, and 0.8 or greater indicating a large effect [14].

### Contrasts

We included three contrasts: online training vs (i) interactive workshops (primary contrast), (ii) taught lectures and, (iii) written/electronic manuals, at one time-point: immediately after the training intervention or as close to completion as possible.

Meta-analyses were performed with Review Manager v5.3 using a random effects model due to expected diversity in population and interventions [9]. Statistical heterogeneity was assessed using the I^2^ statistic and was interpreted as follows: 0% to 40% may not be important; 30% to 60% may represent moderate heterogeneity; 50 to 90% may represent substantial heterogeneity; 75% to 100% high heterogeneity [15]. Additionally, we assessed the effect of methodological quality on effect size in sensitivity analyses. Studies were categorised as ‘low risk’ of bias if they were rated ‘low’ for at least 3 of 5 items on the Cochrane Risk of Bias tool (allocation concealment, blinding of participants, blinding of assessors, intention-to-treat analysis and completeness of outcome data). Disagreements were resolved though discussion.

## Results

**Fig. 1:**
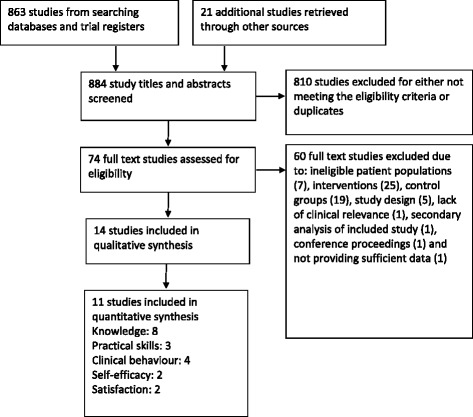
Flow of studies

A total of 884 studies were identified by the literature searches, from which 14 RCTs met the inclusion criteria and 11 provided data for inclusion in the meta-analysis (Fig. 1).

### Description of included trials (Table 1)

From the 14 trials, there were a total of 1089 licensed healthcare professionals recruited from medicine (*n* = 6/14), nursing (*n* = 4/14), mental health counsellors/psychologists (*n* = 2/14), occupational therapy (*n* = 1/14), or multiple disciplines (n = 1/14) (Table 1). The most common comparators were interactive workshops (*n* = 7/14) and taught lectures (*n* = 7/14), and to a lesser degree, written manuals (*n* = 3/14); three of fourteen studies had multiple eligible control groups.

**Table 1:** Description of included trials

Study	Clinical topic	n	Profession	Comparison (duration)	Outcomes^a^					Risk of Bias^b^					
					KW	PS	CB	SE	ST	1	2	3	4	5	H/L
Theoretical knowledge															
Simonsen 2014	Drug dose calculation	183	Nursing	(i) workshop (2 days)	Y			Y	Y^c^	U	H	H	L	L	H
Worm 2013	Respiratory physiology and pulmonology	63	Nursing	(i) lecture (45 mins)	Y					U	H	H	L	L	H
Fordis 2005	Cholesterol management	103	Medical physicians	(i) lecture (90-120 min)	Y^c^		Y		Y^c^	U	H	H	H	L	H
Hugenholtz 2008	Mental health care	74	Occupation health	(i) lecture (30 mins)	Y					U	H	L	H	L	H
Padalino 2007	Quality tools	49	Nursing	(i) lecture (120 mins)	Y					U	H	U	L	L	H
Applied Knowledge/Skills															
Beyea 2007	Particle repositioning manoeuvre	25	Medical residents	(i) lecture (15 mins) (ii) workshop (15 mins)		Y				U	H	L	L	H	H
Chenkin 2008	Ultrasound guided vascular access	21	Medical residents and physicians	(i) lecture (180 mins)	Y	Y			Y^c^	U	H	L	L	L	L
Makinen 2006	Resuscitation and defibrillation	56	Nursing	(i) workshop (240 mins)		Y^c^				U	H	L	L	L	L
Platz 2011	Sonography for trauma	44	Medical	(ii) lecture (60 mins)	Y^c^					U	H	H	H	U	H
Complex interventions															
Bello 2005	Airway management	56	Medical residents	(i) lecture (300 mins)	Y^c^	Y^c^			Y^c^	U	H	L	L	L	L
Dimeff 2009	Dialectical behaviour therapy	174	Mental health (psychologists/counsellors)	(i) manual (n/a) (ii) workshop (1200 mins)	Y	Y	Y	Y	Y	L	H	U	L	H	H
Hearty 2013	Closed reduction and percutaneous pinning	28	Medical physicians	(i) manual (n/a)	Y					U	H	H	L	L	H
Maloney 2011	Exercise for falls prevention	135	Allied health	(i) workshop (420 mins)	Y^c^		Y		Y	L	H	L	H	L	L
Sholomskas 2005	Cognitive behavioural therapy	78	Mental health (psychologists/counsellors)	(i) manual (ii) workshop (1380 mins)	Y	Y^c^	Y			U	H	L	H	L	H

^a^Outcomes are: KW = knowledge, PS = practical skills, CB = clinical behaviour, SE = self-efficacy, and ST = satisfaction. ^b^Risk of Bias items and answers are: 1 = allocation concealment, 2 = blinding of participants, 3 = blinding of outcome assessor, 4 = intention to treat, 5 = loss to follow-up, L = Low risk of bias, H = High risk of bias, U = unclear risk of bias. ^C^outcome data could not be included in meta-analysis

### Intervention description using the TIDieR guidelines

#### Summary of reporting (Table 2)

Of the 12 TIDieR checklist items, all studies reported the health care professional target group, the clinical topic/s and course objectives. The majority of studies reported the intervention duration (learning time) and schedule (length of availability); however, reporting of the course content, type of learning activity such as instruction or feedback, and mode of delivery such as text or video, was insufficient for replication. Moreover, no studies provided information on how to access the online interventions. Details of the course development was provided in only 5 studies and no studies reported any formal pilot testing. Lastly, less than half of the studies reported planned or actual adherence to the online interventions. A description of the study interventions is provided below.

**Table 2:** Description of online interventions for replication

Study	Field (target group)	Learning topic/objectives	Component ingredients and application	No. of sessions	Learning time	Duration available
Theoretical knowledge						
Simonsen 2014	Pharmacology (nurses)	Drug dose calculations: a review of the basic theory of the different types of calculations.	Interactive tests, hints and suggested solutions, access to a collection of tests with feedback on answers	One-off	Two days	2-day course
Worm 2013	Anaesthesiology (nurses)	Lung volume curve and cases related to this and pulmonology.	Clinical cases, pictures and explanation, and presentation	At learners discretion	n/r	2 weeks
Fordis 2005	General practice (medics)	To improve knowledge of and behaviour in line with NIH cholesterol management guidelines	Video and text, interactive cases with feedback, enabling tools (e.g. risk assessment calculator). Participants could also send questions to faculty members via e-mail.	At learners discretion	1.5-2 h	2 weeks
Hugenholtz 2008	Mental health (occupational therapists)	Education on diagnosis, prognosis, and treatment related to mental health and work.	Videos, cases to solve, multiple choice questions, links to relevant literature	One-off session	30 min	n/a
Padalino 2007	Quality tools (nurses)	Quality/process improvement	PowerPoint presentation	One-off session	40 min	Any point in a single night shift
Applied Knowledge/Skills						
Beyea 2007	Family medicine (medics)	Particle repositioning manoeuvre (PRM) for treating benign paroxysmal positional vertigo (BPPV).	Series of slides (text and diagrams) detailing PRM procedure.	One-off session	15 min	n/a
Chenkin 2008	Emergency medicine (medics)	Ultrasound guided vascular access (UGVA) for insertion of central, intravenous, and arterial lines.	Included videos, animations, self-assessment, quizzes, and nonlinear navigation. 2 h practical after online course (no instructors present)	One-off session	1 h	n/a
Makinen 2006	Geriatrics (nursing)	Cardiopulmonary resuscitation and defibrillation (CPR-D)	A case scenario, videos and pictures, links, and questions with feedback.	At learners discretion	15-30 min	2 weeks
Platz 2011	Emergency medicine and surgery (medics)	(i) Ultrasound physics and instrumentation, and (ii) extended focused assessment with sonography for trauma.	Narrated lectures, text, pictures, video clips, 5-min Q&A	One-off session	1 h	n/a
Delivery of Complex interventions						
Bello 2005	Anaesthesiology (medics)	Traditional tracheal intubation and alternative airway management methods.	Text and graphical slides, video demonstrations of each procedure, discussion forum with instructors (3 live sessions)	At learners discretion	5 h	36 h
Dimeff 2008	Mental health (psychologists/counsellors)	Dialectical Behaviour Therapy (DBT), a complex, multi-modal treatment. The training focused solely on the group skills training component of DBT.	Audio and visual material, expert insights, practice exercises, clinical simulations with fictional DBT patients, knowledge checks, printable downloads	At learners discretion	20 h	90 days
Hearty 2013	Orthopaedics (medics)	Performing a closed reduction and percutaneous pinning of a paediatric extension-type supracondylar humeral fracture.	Fully narrated goal based modules that include multimedia such as diagrams, radiographs, animation, and video clips. Self-evaluation tool.	At learners discretion	n/r (12 modules)	n/r
Maloney 2011	Falls prevention (allied health mix)	Exercise prescription for falls prevention	Self-directed reading, formative quizzes, interactive skills-practice with feedback (through uploading digital footage), videos, and reflexive tasks. Also included web based discussions with tutor	At learners discretion	7 h	4 weeks
Sholomskas 2005	Mental health (psychologists/counsellors)	CBT for substance-abuse	Highly text based, multiple choice tests with feedback, case vignettes with exemplary responses	3 months	20 h	duration of trial

### What and how (content, dose, procedures and materials) (Table 2 and Additional file 1)

The topic and complexity of interventions was wide-ranging. Five studies trained HCPs in simple theoretical knowledge, for example, drug dose calculations, and four trained HCPs in the application of knowledge and/or practical skills, for example, cardiopulmonary resuscitation (Table 2). Five studies trained HCPs in complex interventions such as cognitive behavioural therapy. Access to online interventions varied from a single one off session, to on-going access over 90 days, with intervention duration (learning time) ranging from 15 min to 36 h (mean 8.8 h, median 2.5 h). The type of learning activity most commonly reported was the provision of information and instruction to learners, with half of the studies including feedback and practice, and only three studies providing demonstration or additional tools. In terms of delivery mode, the use of text, pictures/animations, and video were most frequently reported.

Of the five studies reporting on the course development, all utilised an interactive design with end-user feedback [16–20]. Six studies reported the technology that was used to build/develop course materials which were: Blackboard (*n* = 1), PowerPoint (*n* = 1), Adobe Flash (*n* = 2), and Moodle (*n* = 2). Two of the fourteen studies offered continuing education credits for completing the online training, although it is not clear if these were certified from an external governing body [17, 21].

### How well (learner adherence and course fidelity)

Only 6 studies reported adherence (mean time spent learning) to the online intervention, 4 of which used a self-reported measure of adherence [16, 17, 19, 22], and 2 used a form of online user analytics [21, 23]. In these studies, adherence was reported as acceptable.

### Sample size and methodological quality

**Fig. 2:**
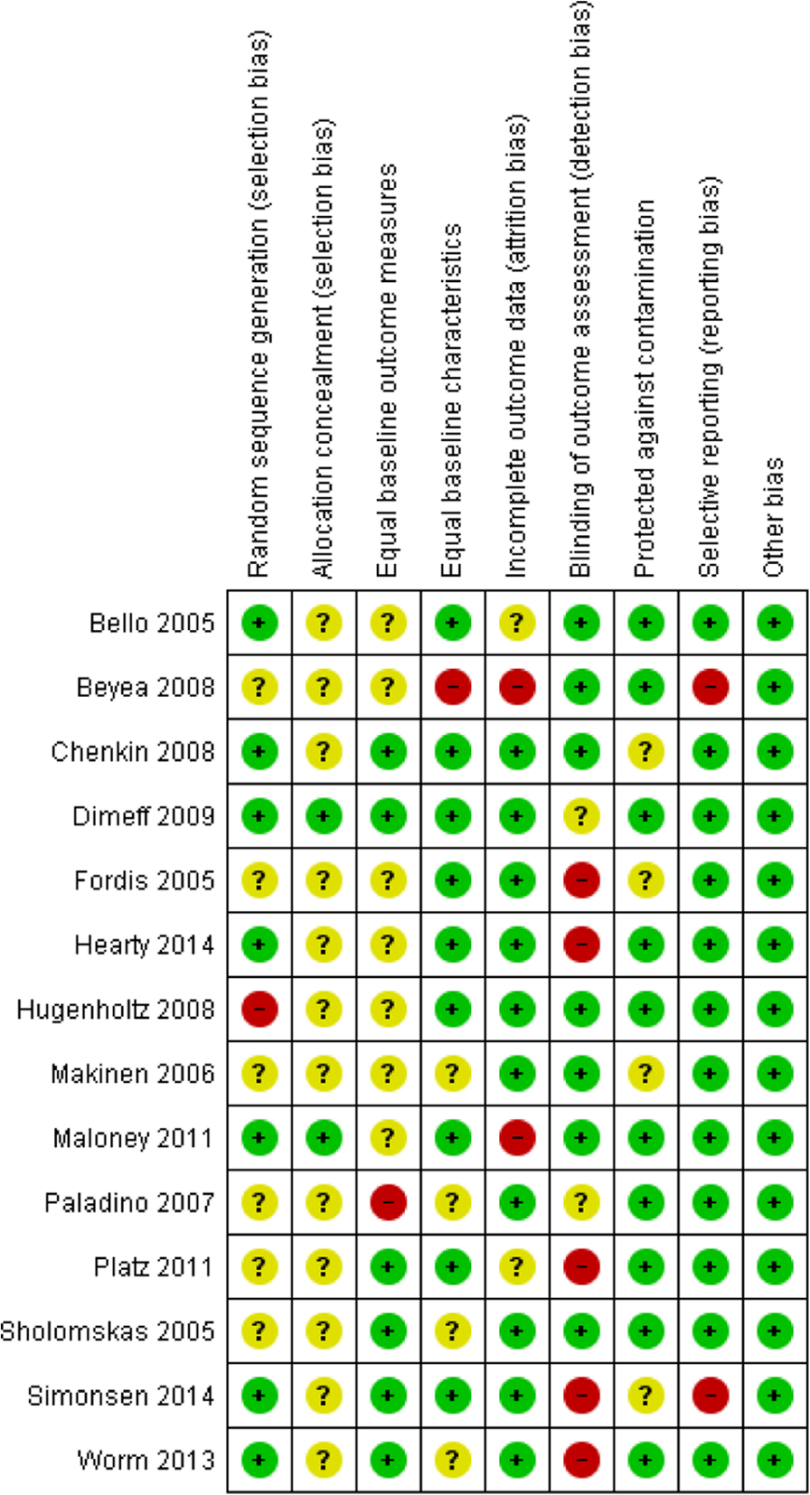
Risk of bias

The majority of sample sizes were small and ranged from 21 to 183. Methodological quality was poor overall with 71% (10/14) of studies classified as having unclear or high risk of bias. Reporting quality was generally poor, leading to judgements of ‘unclear’ risk of bias in 93% (13/14) of studies on at least one of the five items used for classification from the Cochrane Risk of Bias tool (Fig. 2).

### Meta analyses

**Fig. 3:**
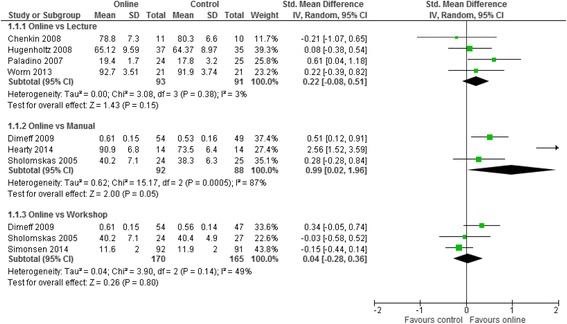
Meta-analysis for knowledge

**Fig. 4:**
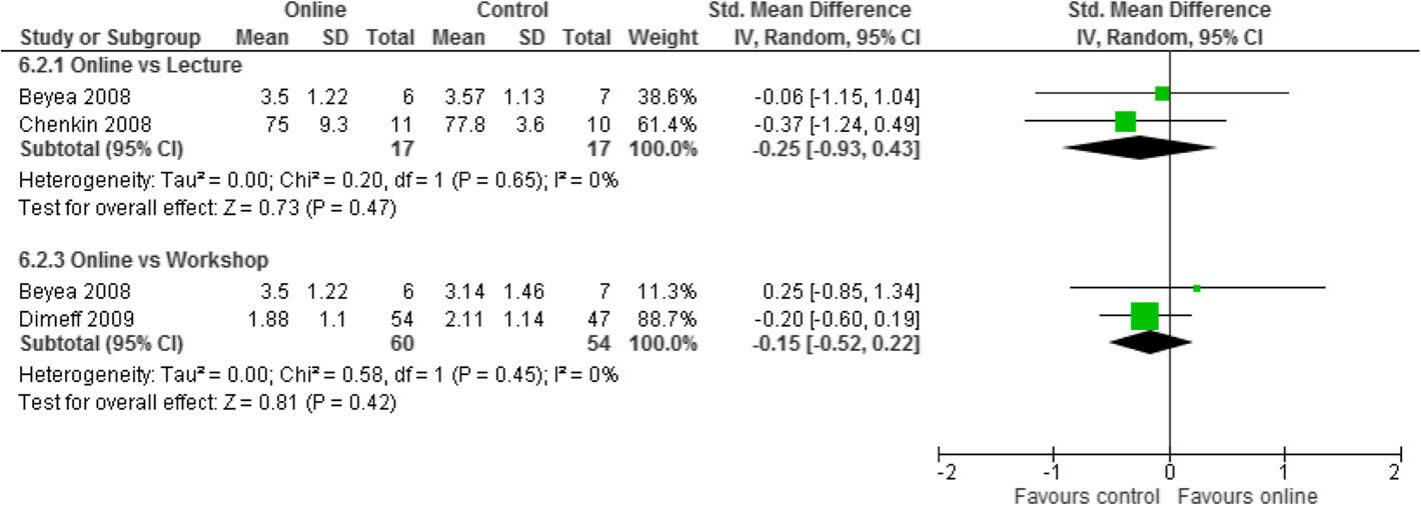
Meta-analysis for practical skills

**Fig. 5:**
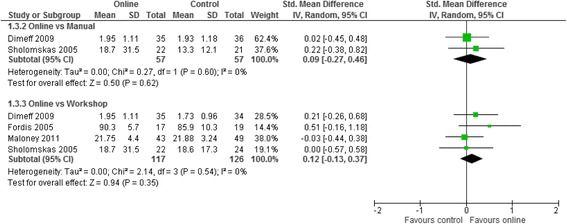
Meta-analysis for clinical behaviour

**Fig. 6:**
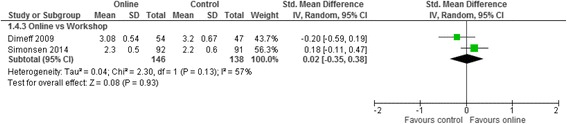
Meta-analysis for self-efficacy

**Fig. 7:**
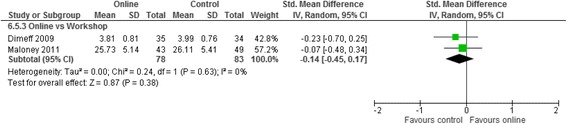
Meta-analysis for satisfaction

Meta analyses for the three contrasts online training vs (i) an interactive workshop (ii) a taught lecture and (iii) a manual at short term for outcomes of knowledge, practical skills, clinical behaviour, self-efficacy, and satisfaction are presented in Figs. 3, 4, 5, 6 and 7. Not all outcomes were available for each contrast and therefore, only outcomes with two more studies are reported below. No studies included patient outcomes. Due to missing data, a number of studies could not be included for the outcomes of (i) knowledge [19, 21, 23, 24], (ii) practical skills [22, 23, 25], and (iii) satisfaction [16, 21, 23]. These individual study results are in line with the findings from the meta-analyses described below. The overall quality of evidence for each outcome and contrast is presented in Table 3; individual study data can be found in Additional files 2 and 3.

**Table 3:** Summary of findings table organised by contrast

Online training methods compared with alternative training methods for licensed health care professionals				
Population: licensed health care professionals Settings: community or health care settings Intervention: online training Comparison: alternative learning methods				
Contrast/Outcome	Standardised Mean Difference (95% CI)	Participants (studies)	Quality (GRADE)	Comments
Face-to-Face Workshop				
Knowledge	SMD 0.04 (−0.28, 0.36)	335 (3)	+ very low^a,b,d^	All assessments were study derived self-assessments. The online learning and workshop interventions ranged between 16 and 20 h. Two further studies assessed knowledge; however, the study data was not suitable to be included in the meta-analysis.
Practical Skills	SMD −0.15 (−0.52, 0.22)	114 (2)	+ very low^a,b,d^	Practical skills were assessed objectively with an objective structured clinical examination (*n* = 1) and a machine to certify the correct procedure had been performed (*n* = 1). Online and workshop training duration was similar within studies but different across studies: 15mins in one study and 20 h in the other. Two further studies assessed practical skills (both using role plays); however, the study data was not suitable to be included in the meta-analysis.
Clinical Behaviour	SMD 0.12 (−0.13, 0.37)	243 (4)	++ low^a,d^	Clinical behavior was assessed with self-reported measures in 3 studies, and with a medical record audit one study. In 3 studies, online and workshop interventions were the same duration and in one study the online training duration was not reported. Between studies, intervention duration ranged from 1.5 to 20 h.
Self efficacy	SMD 0.02 (−0.35, 0.38)	284 (2)	+ very low^a,b,d^	Both studies used self-reported likert scales. Online and workshop intervention duration was the same within each study, but varied between the studies from 20 h to 2 days.
Satisfaction	SMD −0.14 (−0.45, 0.17)	161 (2)	+ very low^a,b,d^	One study used a self-reported likert scale and the other study did provide any information on the measurement tool. Online and workshop intervention duration was the same within each study, but varied between the studies from 7 to 20 h. A third study assessed satisfaction; however, the study data was not suitable to be included in the meta-analysis.
Manual				
Knowledge	SMD 0.99 (0.02, 1.96)	180 (3)	+ very low^a,b,d^	All assessments were study derived self-assessments. The online training was similar in two studies (20 h) and not reported in the other. No information was provided on length of time spent reading the manual in any study.
Practical Skills	–	–	No evidence	–
Clinical Behaviour	SMD 0.09 (−0.27, 0.46)	114 (2)	+ very low^a,c,d^	Both studies used self-reported measures of behavior. The online training intervention was the same duration in both studies (20 h).
Self efficacy	–	–	No evidence	–
Satisfaction	–	–	No evidence	–
Lecture				
Knowledge	SMD 0.22 (−0.08, 0.51)	184 (4)	+ very low^a,b,d^	All assessments were study derived self-assessments. The online learning and lecture interventions were different across studies ranging from 15 min to 20 h. In all but two studies, intervention duration was similar within each study. A fifth study (intervention duration 36 h) assessed this outcome but did not provide any usable data for analysis.
Practical Skills	SMD −0.25 (−0.93, 0.43)	34 (2)	+ very low^a,c,d^	Practical skills were assessed objectively with a series of objective structured clinical examinations (*n* = 1) and a machine to certify the correct procedure had been performed (*n* = 1). The duration of online and lecture interventions was the same within studies, and similar across studies, both being ≤1 h. A third study assessed this outcome but did not provide any usable data for analysis.
Clinical Behaviour	–	–	No evidence	–
Self efficacy	–	–	No evidence	–
Satisfaction	–	–	No evidence	Two studies assess this outcome with a self-report measure. However, the data provided was not suitable for inclusion in a meta-analysis.

^a^Downgraded due to risk of bias, ^b^downgraded due to inconsistency, ^c^downgraded due to indirectness, ^d^downgraded due to imprecision

### Online training vs interactive workshop

Seven RCTs [17, 19, 21, 22, 25–27] compared online training to a workshop, which ranged in duration from 15 mins to 20 h. The most commonly assessed outcome was knowledge (71%), measured with non-validated self-report questionnaires developed specifically within each study. Clinical behaviour and practical skills were assessed in 57% of the studies. Clinical behaviour was assessed with self-reported measures (*n* = 3) and an objective audit of medical records (*n* = 1), while practical skills were assessed with an objective structured clinical examination (OSCE; *n* = 2), role play (*n* = 1), and a machine to certify the correct procedure had been performed (*n* = 1). Self-efficacy and satisfaction were assessed to a lesser degree (self-efficacy: 29%, satisfaction: 43%). Two studies assessing knowledge and satisfaction [19, 21], and two studies assessing practical skills [22, 25] were precluded from meta-analyses due to unusable data.

For clinical behaviour (4 RCTs, *n* = 280), we found low quality evidence that there was no difference between online training and an interactive workshop [SMD 0.12; 95% CI: -0.13, 0.37]. For knowledge (3 RCTs, *n* = 335), practical skills (2 RCTs, *n* = 114), self-efficacy (2 RCTs, *n* = 284) and satisfaction (RCTs n = 2, *n* = 193), we found very low quality evidence that there was no difference between participating in online training or an interactive workshop [Knowledge: SMD 0.04; 95% CI:-0.28, 0.36; practical skills: SMD -0.15; 95% CI: -0.52, 0.22; Self-efficacy: SMD 0.02; 95% CI: -0.35, 0.38; satisfaction: SMD -0.14; 95% CI: -0.45, 0.17]. The summary effect sizes were judged to be of low or very low quality based on high risk of bias, inconsistency and imprecision of the results. Results from individual studies precluded from the meta-analyses showed no significant between group differences for any of their outcomes (knowledge, practical skills and satisfaction), with the exception of a single study [25], who found a statistically significant effect in favour of the workshop for practical skills.

### Online training vs taught lecture

Seven studies [16, 20, 23, 24, 26, 28, 29] compared online training with a taught lecture, ranging in duration from 15 min to 20 h. The majority of studies assessed knowledge (86%) with study specific measures, while three studies (43%) used OSCEs to assess practical skills. Satisfaction was assessed in two studies (29%), while clinical behaviour and self-efficacy were not assessed by studies in this contrast. Within this contrast, three studies were precluded from meta-analyses due to unsuitable data: one that assessed knowledge and practical skills [23], and two that assessed satisfaction [16, 23]. A further study (Platz) was excluded from the meta-analysis for knowledge since they presented change scores only.

For Knowledge (4 RCTS, *n* = 184) and practical skills (2 RCTs, *n* = 34) we found very low quality evidence that there was no difference between online training and taught lectures (knowledge: SMD 0.22; 95% CI: -0.08, 0.51, practical skills: SMD -0.25; 95% CI: -0.93, 0.43). The summary effect estimates were judged to be of very low quality due to high risk of bias, inconsistency, and imprecision of the results.

Results from individual studies precluded from the meta-analyses showed no significant between group differences for any of their outcomes (knowledge, practical skills, and satisfaction) with the exception of satisfaction in a single study [23], which favoured the online group.

### Online training vs written manual

Three studies [17, 22, 30] compared online training to a written manual. All studies (100%) assessed knowledge with study-derived measures, and two studies (67%) assessed clinical behaviour with a self-reported measure. No studies assessed practical skills, self-efficacy, or satisfaction in this contrast. For knowledge (3 RCTs, *n* = 180), we found very low quality evidence that online training was more effective than a manual (SMD 0.99; 95% CI: 0.02, 1.96). For clinical behaviour, we found very low quality evidence that online training was no different to using a written manual (SMD 0.09; 95% CI: -0.27, 0.46). As with the previous contrasts, the summary effect estimates were judged to be of very low quality due to high risk of bias, inconsistency, and imprecision of the results.

### Sensitivity analysis

Four of fourteen studies were categorised as low risk of methodological bias. However, there were insufficient numbers of studies within each contrast to enable examination of effects for studies with low risk of bias only.

## Discussion

### Statement of principal findings

This is the first systematic review to evaluate online versus alternative learning methods since 2008. We identified 11 new studies published since 2008 and built on this prior review by synthesising results in a more focused population and by providing more detailed descriptions of study interventions, providing greater context to our results. To our knowledge, this is the first systematic review to synthesise evidence from RCTs on the effectiveness of online versus alternative methods for training licensed HCPs in clinical interventions. The trials in this review studied the effectiveness of online training across a range clinical topics with varying degrees of complexity. Overall, the summary effect sizes tended to indicate that there was likely little difference on outcomes of knowledge and clinical behaviour between using online training and alternative forms of training including face-to-face workshops, taught lectures or manuals. However, the quality of evidence for all comparisons was assessed as either low or very low. Additionally, there were too few studies to draw any conclusions on the effects of online training for practical skills, self-efficacy, and satisfaction across all contrasts. Therefore, while we believe the results support the potential for online training to be as effective as alternative methods, we recommend interpreting the effect estimates with caution; bearing in mind that the observed inconsistency and imprecision among studies introduces uncertainty regarding our conclusions.

### Outcome significance in relation to other research

While our effect estimates have a degree of uncertainty, the overall findings are in line with the largest systematic review in this field, published in 2008 [2]. Cook et al. pooled 76 non-randomised and randomised studies in meta-analyses and found no significant difference between online training and alternative methods for training health care students/graduates in the outcomes of knowledge, skills, behaviour and satisfaction [2]. Similarly to our current review, previous reviews have reported inconsistent effect sizes in varying directions and magnitude [2, 31]. Our review has strengthened the literature on the effectiveness of online training compared to alternative interventions by only including RCTs and by using the GRADE approach to interpret our findings. As a result, our interpretation of the results from this meta-analysis are in contrast to those drawn in previous reviews [2, 31], which have advocated equivalence in online and alternative training methods, advising against future research into such comparisons. Instead, we argue that there is only low or very low quality evidence in this field, and thus there is a need for future work here*.* Our interpretations of the need for future robust RCTs in this field may be contested by some in the literature due to (i) difficulties in establishing what is actually responsible for the observed effect, (ii) the dilution of effects due to the pragmatic nature of such large RCTs, and (iii) a lack of adequate control interventions (for example, Cook 2005 [32]). However, since educational interventions are by definition complex interventions, the notion that we are often unable to ascertain which part of the intervention (if not the combination of all parts) is responsible for the resulting effects is not unfamiliar. This is somewhat mitigated in healthcare interventions when we are able to measure a number of potential mediating variables, allowing some post-trial exploration of variance. Whilst this proves more difficult in the context of online learning, factors such as engagement, satisfaction, and usage could be measured and later explored. Importantly, detailed descriptions of interventions would allow exploration of any observed heterogeneity. Additionally, we appreciate that given the pragmatic nature of the RCTs included in our current review, the intervention effects are likely to be small. Here it is the interpretation of the effect size that is important, given that while a pragmatic trial may find only a small intervention effect, due to the pragmatic nature of the trial, that small effect may be clinically/educationally important. Moreover, due to the small sample sizes used, existing trials may be underpowered to detect any small but potentially important effects. Thus, rather than concluding that these challenges render the RCT method inadequate, we feel that there is still a strong need for robust, pragmatic, and adequately powered RCTs, with detailed descriptions of interventions, in the field of educational research.

### Limitations

Our review used rigorous methods in accordance with Cochrane and PRISMA guidelines including a sensitive search strategy in multiple databases, and having two authors independently complete all study processes (screening, data checking, and risk of bias assessment). While we conducted a comprehensive search, it is possible that not all relevant RCTs were identified. We increased the robustness of our findings by only including RCT designs, thereby excluding observational study designs. Evidence has shown that observational study designs can offer important and unbiased findings in clinical and education research [33, 34]. However, while we appreciate that some non-randomised studies can provide unbiased effects, this is not true of all non-randomised studies and more importantly, the extent of bias cannot be readily assessed.

We improved contextualisation of current evidence through more specific selection criteria (population and intervention) and increased our level of intervention information to align with recommendations from the TIDieR Guidelines. Despite these strengths, our observed effect sizes were imprecise with wide confidence intervals, as indicated by the GRADE ratings, and thus the results from the meta-analyses should be interpreted cautiously. Furthermore, as control interventions often differed from online interventions in ways other than the mode of delivery (for example, only including personalised feedback in the online arm), we cannot determine whether the difference in effect was due to the online method of delivery, or differences in other factors, such as the content provided or the level of engagement.

The majority of studies provided outcome data immediately after training only. Thus, we do not know whether either method, online or alternate forms of training, were actually effective on their own without comparison to baseline values. Furthermore, all studies employed study-specific outcome measures with unknown clinometric properties limiting our ability to determine whether the observed large confidence intervals contained effect sizes of educational importance. Due to a lack of high quality studies in each contrast, we were unable to assess the influence of methodological quality on our effect estimates. Further, we could not assess publication bias by funnel plot asymmetry due to the small number of studies in the meta-analyses. Lastly, we made a pragmatic decision to limit our search from the year 2000 onwards. However, from searching the 201 included studies in Cook et al.’s comprehensive review, there were no studies published prior to the year 2000 that would have been eligible for inclusion in this review [2].

### Implications

In terms of providing HCPs with knowledge and achieving desired clinical behaviour, the results from this review suggest that online training may be as effective as alternative methods. Thus, factors such as the availability of resources, expertise, and desired reach of an intervention may govern which training method is optimal in any given scenario. Due to the small number of studies assessing practical skills, satisfaction, and self-efficacy, we cannot provide useful or confirmatory evidence to recommend using online methods for training licenced HCPs in clinical interventions until a robust, direct comparison study has been conducted.

### Practical implications for educators: consideration of pedagogy and approaches in the health sciences

The increasing reliance and use of technology is impacting on the teaching approaches used by health educators. For example, the collaborative learning approach has evolved through technology and can now be achieved through shared learning spaces and with various methods such as video and blogs. Additionally, technology has expanded learning options, enabling learners to engage with learning through multiple methods such as open access courses, and allowing them to choose how, when and where they learn, facilitating independence. In practice, we found that the online learning interventions in this review combined multiple technological approaches and seldom reported any pedagogical rationale for their use. Moreover, studies did not include process evaluations to ascertain if the technological approaches were the effective components of the intervention. Thus, we do not know which approaches are optimal for any given context or task. Future research should specify what pedagogical approach they are using and how they plan to evaluate the approach to provide evidence of usefulness of specific intervention features. Where possible, studies should compare different pedagogical approaches in head to head comparisons.

### Future work

We observed inconsistent results due to varying directions of effect estimates across studies, with some studies favouring online and other studies favouring the alternative training methods. The inconsistency in results could be due to a number of differences in the included populations and interventions (topic, complexity, components). It is plausible that online training may be better or worse than other methods depending on these factors; however, the limited number of studies, and poor descriptions of clinical topics and intervention components precluded exploration of the impact of clinical heterogeneity on outcome. Thus, to improve our understanding on the effectiveness of online compared to alternative training methods for licensed health care professionals, we need robust and adequately powered RCTs with well described intervention and control arms. We therefore recommend that studies follow the TIDieR Guidelines for reporting intervention information and include detailed information on intervention dose and content, as well as assessing participant compliance. It is important to ensure that comparison interventions are designed to be similar in potential confounding factors such as duration and content. With regards to outcomes, this evidence base could be improved with consistent use of Kirkpatrick’s outcome hierarchy to ensure all aspects of training are assessed [35, 36].

We also recommend that future studies compare the effectiveness of different online learning approaches through tightly-controlled experiments with factorial designs in order to optimise the features of online interventions to increase adherence and retention of knowledge and/or skills. Lastly, future trials should explore the cost effectiveness of online versus alternative methods of training.

### What this study adds

Our small and imprecise pooled effect sizes are similar to those found in Cook et al.’s review conducted in 2008, highlighting that study quality has not improved in this field over time and thus supporting our conclusions that robust and adequately powered RCTs are needed to progress this field of study. This study builds on Cook et al.’s meta-analysis by providing detailed information about the included study interventions to facilitate replication and allow future educators to identify interventions of relevance to them. This is particularly important when considering that many online learning interventions may not be generalisable to other contexts/fields due to the specificity of the subject matter.

## Conclusion

While we found very low quality evidence that online methods may be as effective as alternative methods for training licenced HCPs in clinical interventions for the outcomes of knowledge and clinical behaviour, the confidence intervals around our effect sizes were large and could encompass important differences in effectiveness. The evidence provided in this review was limited by trials with small sample sizes, poor methodological quality, missing outcomes, and inadequate reporting of online interventions. To recommend online over alternative training methods in this population, more robust, adequately powered RCTs are needed with detailed intervention descriptions.

## Additional files

Additional file 1: Reported intervention components. (DOCX 98 kb)

Additional file 2: All study data. Contains data from all studies including those not suitable for inclusion in the meta-analyses. (XLSX 25 kb)

Additional file 3: Meta analysis data. Contain only the data from studies used in the meta-analyses. (XLSX 15 kb)

## Abbreviations

CI: Confidence interval
GRADE: Grading of Recommendations, Assessment, Development and Evaluations
HPC/s: Health Care Professionals
MD: Mean difference
OSCE: Objective Structured Clinical Examinations
PRISMA: Preferred Reporting Items for Systematic Reviews and Meta-Analyses
RCT/s: Randomised controlled trial
TIDieR: Template for Intervention Description and Replication
UK: United Kingdom
