# Supplementary material for: A systematic review and meta-analysis of online versus alternative methods for training licensed health care professionals to deliver clinical interventions
Source: BMC Med Educ. 2017 Nov 23;17:227. doi: 10.1186/s12909-017-1047-4 (PMC5701457; doi:10.1186/s12909-017-1047-4)
Supplement: Supplementary file 1 — Reported intervention components. (DOCX 98 kb) [file 12909_2017_1047_MOESM1_ESM.docx]

**Additional file 1. Reported intervention components**

| **Study** | **Online course component ingredients and application** | **Information** | **Instruction** | **Demonstration** | **Practice** | **Feedback** | **Additional objects / tools** |
| --- | --- | --- | --- | --- | --- | --- | --- |
| **Simonsen** | Review of theory, examples and exercises, interactive tests, hints and suggested solutions, access to a collection of tests with feedback on answers |  |  |  |  |  |  |
| **Worm** | Clinical cases, pictures and explanation, and presentation |  |  |  |  |  |  |
| **Fordis** | Video and text, interactive cases with feedback, enabling tools (e.g. risk assessment calculator). Participants could also send questions to faculty members via e-mail. |  |  |  |  |  |  |
| **Hugenholtz** | Cases to solve, multiple choice questions, links to relevant literature |  |  |  |  |  |  |
| **Padalino** 2007 | PowerPoint presentation |  |  |  |  |  |  |
| **Makinen** | Begins with a case scenario, videos and pictures, links, and questions with feedback. |  |  |  |  |  |  |
| **Beyea** 2007 | Series of slides (text and diagrams) detailing PRM procedure. |  |  |  |  |  |  |
| **Chenkin** 2008 | Included videos, animations, self-assessment, quizzes, and nonlinear navigation. 2 hour practical after online course (no instructors present) |  |  |  |  |  |  |
| **Bello** 2005 | Text and graphical slides, video demonstrations of each procedure, discussion forum with instructors (3 live sessions) |  |  |  |  |  |  |
| **Hearty** | Fully narrated goal based modules that include multimedia such as diagrams, radiographs, animation, and video clips. Self-evaluation tool. |  |  |  |  |  |  |
| **Platz** | Narrated lectures, text, pictures, video clips, 5-minute Q&A |  |  |  |  |  |  |
| **Dimeff** | Audio and visual material, expert insights, practice exercises, clinical simulations with fictional DBT patients, knowledge checks, printable downloads |  |  |  |  |  |  |
| **Maloney** | Self-directed reading, formative quizzes, interactive skills-practice with feedback (through uploading digital footage), videos, and reflexive tasks. Also included web based discussions with tutor |  |  |  |  |  |  |
| **Sholomskas** | Highly text based, multiple choice tests with feedback, case vignettes with exemplary responses |  |  |  |  |  |  |
| **Mode:**  Text Audio Pictures and/or animations Video Access to person | | | | | | | |
